# Long-term safety, tolerability, and efficacy of efgartigimod (ADAPT+): interim results from a phase 3 open-label extension study in participants with generalized myasthenia gravis

**DOI:** 10.3389/fneur.2023.1284444

**Published:** 2024-01-17

**Authors:** James F. Howard, Vera Bril, Tuan Vu, Chafic Karam, Stojan Peric, Jan L. De Bleecker, Hiroyuki Murai, Andreas Meisel, Said R. Beydoun, Mamatha Pasnoor, Antonio Guglietta, Benjamin Van Hoorick, Sophie Steeland, Caroline T’joen, Kimiaki Utsugisawa, Jan Verschuuren, Renato Mantegazza, the ADAPT+ Study Group

**Affiliations:** ^1^Department of Neurology, The University of North Carolina at Chapel Hill, Chapel Hill, NC, United States; ^2^Ellen and Martin Prosserman Centre for Neuromuscular Diseases, University Health Network, University of Toronto, Toronto, ON, Canada; ^3^Department of Neurology, University of South Florida Morsani College of Medicine, Tampa, FL, United States; ^4^Penn Neuroscience Center-Neurology, Hospital of the University of Pennsylvania, Philadelphia, PA, United States; ^5^Neurology Clinic, University Clinical Center of Serbia, Faculty of Medicine, University of Belgrade, Belgrade, Serbia; ^6^Department of Neurology and Neuromuscular Reference Center, Ghent University Hospital, Ghent, Belgium; ^7^Department of Neurology, School of Medicine, International University of Health and Welfare, Narita, Japan; ^8^Department of Neurology, Charité – Universitätsmedizin Berlin, Berlin, Germany; ^9^Department of Neurology, Keck School of Medicine, University of Southern California, Los Angeles, CA, United States; ^10^Department of Neurology, University of Kansas Medical Center, Kansas City, KS, United States; ^11^argenx, Ghent, Belgium; ^12^Department of Neurology, Hanamaki General Hospital, Hanamaki, Japan; ^13^Department of Neurology, Leiden University Medical Center, Leiden, Netherlands; ^14^Department of Neuroimmunology and Neuromuscular Diseases, Fondazione Istituto Carlo Besta, Milan, Italy

**Keywords:** efgartigimod, myasthenia gravis, neonatal Fc receptor, FcRn, IgG recycling, antibody fragment, autoantibody reduction, neonatal Fc receptor antagonist

## Abstract

**Objective:**

ADAPT+ assessed the long-term safety, tolerability, and efficacy of efgartigimod in adult participants with generalized myasthenia gravis (gMG).

**Methods:**

ADAPT+ was an open-label, single-arm, multicenter, up to 3-year extension of the pivotal phase 3 ADAPT study. Efgartigimod was administered in treatment cycles of 4 intravenous infusions (one 10 mg/kg infusion per week). Initiation of subsequent treatment cycles was individualized based on clinical evaluation. Safety endpoints included incidence and severity of adverse events. Efficacy endpoints assessed disease severity using Myasthenia Gravis-Activities of Daily Living (MG-ADL) and Quantitative Myasthenia Gravis (QMG) scores.

**Results:**

As of January 2022, 151 participants had rolled over to ADAPT+ and 145 had received ≥1 dose of efgartigimod, of whom, 111 (76.6%) were AChR-Ab+ and 34 (23.4%) were AChR-Ab−. Mean study duration (treatment plus follow-up) was 548 days, and participants received up to 17 treatment cycles, corresponding to 217.6 participant-years of exposure. In the overall population, 123 (84.8%) participants reported ≥1 treatment-emergent adverse event; most frequent were headache (36 [24.8%]), COVID-19 (22 [15.2%]), and nasopharyngitis (20 [13.8%]). Clinically meaningful improvement (CMI) in mean MG-ADL and QMG scores was seen as early as 1 week following the first infusion across multiple cycles in AChR-Ab+ and AChR-Ab− participants. Maximal MG-ADL and QMG improvements aligned with onset and magnitude of total IgG and AChR-Ab reductions. For AChR-Ab+ participants at any time point in each of the first 10 treatment cycles, more than 90% had a maximum reduction of ≥2 points (CMI) in MG-ADL total score; across the 7 cycles in which QMG was measured, 69.4% to 91.3% of participants demonstrated a maximum reduction of ≥3 points (CMI) in QMG total score. Many participants demonstrated improvements well beyond CMI thresholds. In AChR-Ab+ participants with ≥1 year of combined follow-up between ADAPT and ADAPT+, mean number of annualized cycles was 4.7 per year (median [range] 5.0 [0.5–7.6]).

**Conclusion:**

Results of ADAPT+ corroborate the substantial clinical improvements seen with efgartigimod in ADAPT and support its long-term safety, tolerability, and efficacy, as well as an individualized dosing regimen for treatment of gMG.

**Clinical trial registration:**

https://classic.clinicaltrials.gov/ct2/show/NCT03770403, NCT03770403.

## 1 Introduction

Generalized myasthenia gravis (gMG) is a rare, chronic, and potentially life-threatening neuromuscular autoimmune disease. It is characterized by debilitating, and potentially severe, weakness of ocular, facial, bulbar, respiratory, and limb muscles (1, 2). MG is caused by pathogenic immunoglobulin G (IgG) autoantibodies binding to components of the neuromuscular junction, disrupting neuromuscular transmission (3). Using conventional assays, approximately 85% of patients with gMG have been shown to have identifiable IgG autoantibodies targeting skeletal muscle acetylcholine receptors (AChRs); a smaller proportion have autoantibodies targeting muscle-specific tyrosine kinase (MuSK) or lipoprotein receptor–related protein 4 (LRP4) (4). The remaining ~10% without identifiable autoantibodies may benefit from more-sensitive cell-based assays (5, 6).

Treatments used for MG, such as corticosteroids and nonsteroidal immunosuppressant therapies (NSISTs), broadly suppress the immune system but do not selectively target IgG autoantibodies, which are central to gMG pathophysiology (7). NSISTs frequently provide insufficient symptom relief and have a delayed (1–2 years) onset of effect, and corticosteroids have burdensome adverse effects that can limit their use, as well as increased healthcare resource utilization costs (7–10). The more-recent complement inhibitors target only 1 of the 3 pathogenic pathways triggered specifically by AChR autoantibodies (11). Ultimately, there remains a significant unmet need for gMG treatment options that are effective, well tolerated, targeted toward autoantibodies, and can be used in a broad population of patients (3, 4, 12).

Efgartigimod, which blocks the neonatal Fc receptor (FcRn), is being studied as a potential treatment in several IgG-mediated autoimmune diseases. FcRn recycles IgG, including pathogenic IgG autoantibodies, resulting in a higher concentration and longer half-life than that of immunoglobulins not recycled by FcRn (13). FcRn also promotes transcytosis of IgG into tissue and recycles albumin, which binds at a distinct site from the IgG-binding site (13, 14). Efgartigimod is a human IgG1 antibody Fc fragment, a natural ligand of FcRn that binds to the normal IgG binding site at a site distinct from albumin (13). Efgartigimod was engineered for increased affinity to FcRn with retention of characteristic pH-dependent binding, which increases binding in endosomal conditions to outcompete endogenous IgG (13, 15). Efgartigimod prevents recycling of IgG without impacting its production and selectively reduces all IgG subtypes, including pathogenic IgG autoantibodies, without affecting other immunoglobulins (ie, IgM, IgA, IgE, IgD) (13, 16). Additionally, it does not prevent an immune response against vaccination (17) and has been shown neither to reduce serum albumin nor increase cholesterol levels (13, 18, 19).

Results from the randomized, placebo-controlled, phase 3 ADAPT study (NCT03669588) (20) demonstrated that efgartigimod was well tolerated and efficacious in adults with gMG (18). Intravenous (IV) efgartigimod received approval in the US and EU for adult patients with AChR antibody–positive (AChR-Ab+) gMG and was approved in Japan regardless of antibody status (21–23).

ADAPT+ (NCT03770403) (24) was an open-label extension of the pivotal ADAPT study (18), assessing long-term safety, tolerability, and efficacy of efgartigimod. Here we present results from the final interim data analysis (cutoff date: January 31, 2022) of ADAPT+.

## 2 Methods

### 2.1 Study design

**Figure 1:**
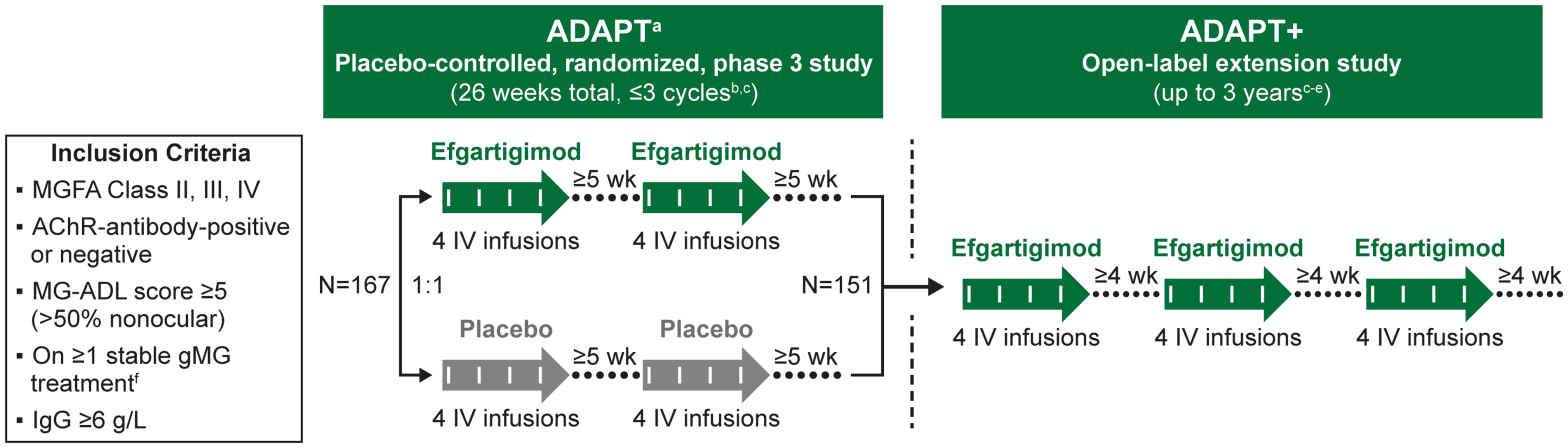
Study design. AChEI, acetylcholinesterase inhibitor; AChR, acetylcholine receptor; gMG, generalized myasthenia gravis; HCP, healthcare provider; IgG, immunoglobulin G; IV, intravenous; MG-ADL, Myasthenia Gravis-Activities of Daily Living; MGFA, Myasthenia Gravis Foundation of America; NSIST, nonsteroidal immunosuppressive therapy. In ADAPT+ efgartigimod was administered in treatment cycles comprising 4 once-weekly IV infusions (10 mg/kg over 1 h) per cycle on days 1, 8, 15, and 22 of each cycle, with follow-up of at least 4 weeks. ^a^ Participants who required retreatment but were unable to complete a treatment cycle within the time frame of ADAPT were eligible to be rolled over to ADAPT+. ^b^ Subsequent cycles initiated if ≥5 weeks from last infusion, MG-ADL score improvement <2 points from baseline, and MG-ADL total score ≥5 (>50% due to nonocular items). ^c^ Participants requiring rescue therapy in ADAPT and in year 1 of ADAPT+ were discontinued from the study. Participants requiring rescue in years 2 and 3 of ADAPT+ were not discontinued. ^d^ Subsequent cycles initiated in the first year of ADAPT+ if ≥4 weeks from last infusion of previous cycle, MG-ADL score improvement <2 points from cycle baseline, and MG-ADL total score ≥5 (>50% due to nonocular items). ^e^ Subsequent cycles were initiated in years 2 and 3 of ADAPT+ if ≥4 weeks from last infusion of previous cycle, at the discretion of the investigator. ^f^ AChEI, corticosteroid, and/or NSIST. HCPs could not change concomitant therapies in ADAPT; HCPs could change concomitant therapies in ADAPT+.

ADAPT+ was an open-label, single-arm, multicenter, 3-year extension of the pivotal ADAPT study. It was conducted at 51 sites worldwide. Efgartigimod (10 mg/kg) was administered in treatment cycles of 4 weekly IV infusions (1 infusion per week). Participants were assessed at each infusion visit, every 30 (±2) days during the first year of the study, and every 90 (±7) days after the first year (Figure 1).

The number of treatment cycles a participant received was individualized based on clinical evaluation. In the first year of ADAPT+, subsequent cycles were initiated ≥4 weeks after the last infusion of the previous cycle if a participant had a Myasthenia Gravis-Activities of Daily Living (MG-ADL) total score ≥5 (with >50% of the score from non–ocular-related items) and no longer had a clinically meaningful improvement (CMI; defined as a ≥2-point improvement in total MG-ADL score) compared with the cycle baseline. After the first year of participation, the only requirement was a minimum of 4 weeks having elapsed since the last efgartigimod infusion of a previous cycle and clinical judgment of the physician that the next cycle would be of benefit.

### 2.2 Participants

Participants who completed the ADAPT study, or who were eligible for a treatment cycle but could not complete the cycle by week 26 of ADAPT, were eligible to enter ADAPT+. Because participants came directly into ADAPT+ from ADAPT, they were on a stable dose of ≥1 MG medication. ADAPT required participants to be on a stable dose of MG medication before study enrollment: acetylcholinesterase (AChE) inhibitors, no dose escalation in the 2 weeks before study screening; corticosteroids, treatment initiation at least 3 months prior, without dose changes in the month before screening; NSISTs, treatment initiation at least 6 months prior, without dose changes in the 3 months before screening. Participants could not change medication while in the ADAPT study. See Supplementary Table 1 for a complete list of inclusion/exclusion criteria. Additionally, participants who completed ≥1 treatment cycle and ≥1 year of ADAPT+ were eligible to roll over to the open-label study of subcutaneously (SC) administered efgartigimod PH20 (coformulated with recombinant human hyaluronidase PH20 [ADAPT-SC+; NCT04818671]) (25).

ADAPT+ was conducted in accordance with principles of the Declaration of Helsinki, the International Council for Harmonisation on Good Clinical Practice, and other applicable local ethical and legal requirements. The study protocol, amendments, and informed consent forms were approved by institutional review boards, ethics committees, and regulatory agencies. Study data are presented in accordance with CONSORT guidelines (26).

### 2.3 Procedures

The primary objective was to investigate the long-term safety and tolerability of efgartigimod in AChR-Ab+ participants. The secondary objective was to evaluate long-term safety and tolerability in the overall population, which included both AChR-Ab+ and AChR-Ab–negative (AChR-Ab−) participants. Throughout the study, endpoints included evaluation of the following variables: incidence and severity of treatment-emergent adverse events (TEAEs), serious AEs, and abnormalities in vital signs, electrocardiograms, and laboratory assessments.

Efficacy assessments evaluated disease severity in AChR-Ab+ participants, AChR-Ab− participants, and in the overall study population using MG-ADL and Quantitative Myasthenia Gravis (QMG) total scores for each treatment cycle. A CMI in MG-ADL total score was a reduction of ≥2 points; a CMI in QMG score was a reduction of ≥3 points (27, 28). MG-ADL assessments were captured for up to 17 cycles, but data reported are only through cycle 10 due to limited sample size in subsequent cycles. QMG was only required to be captured during the first year of ADAPT+ (up to 7 treatment cycles).

Pharmacodynamic assessments included levels of total IgG and IgG subtypes (IgG1, IgG2, IgG3, and IgG4), AChR autoantibodies in participants who were AChR-Ab+, and MuSK autoantibodies in participants who were MuSK antibody–positive (MuSK-Ab+). Pharmacodynamic assessments were only required to be captured during the first year of ADAPT+ (up to 7 treatment cycles). Immunogenicity assessments included antidrug antibody (ADA) analyses. Vaccination (except for live/live-attenuated vaccines) was allowed 48 h before or 48 h after efgartigimod administration in ADAPT+; in participants who provided additional consent, vaccine-induced IgG responses were studied and reported elsewhere (17).

### 2.4 Statistical analysis

The analyses were performed on all participants who received ≥1 dose of efgartigimod. Safety was summarized using descriptive statistics. TEAEs were coded using Medical Dictionary for Regulatory Activities central coding dictionary (version 24.1) and graded using the National Cancer Institute Common Terminology Criteria for Adverse Events (version 5.0).

Summary statistics of absolute values and changes from cycle baseline were calculated for MG-ADL and QMG total scores for the AChR-Ab+ population, AChR-Ab− population, and combined safety (hereafter referred to as overall) population. Mean and median annualized cycles per year were analyzed in AChR-Ab+ participants with at least 1 year of exposure to efgartigimod across ADAPT and/or ADAPT+. Categories of annualized cycles per year were rounded to the nearest whole cycle. The mean time between cycles, defined as the last infusion of the previous cycle to first infusion of the next cycle, was calculated across each AChR-Ab+ participant’s cycles and grouped into 1 of 3 categories: <6 weeks, 6 to 9 weeks, and ≥9 weeks. Statistical analyses were performed using a statistical analysis package (SAS®) (SAS Institute, Cary, NC, USA) version 9.4 or later.

## 3 Results

### 3.1 Participant population

**Figure 2:**
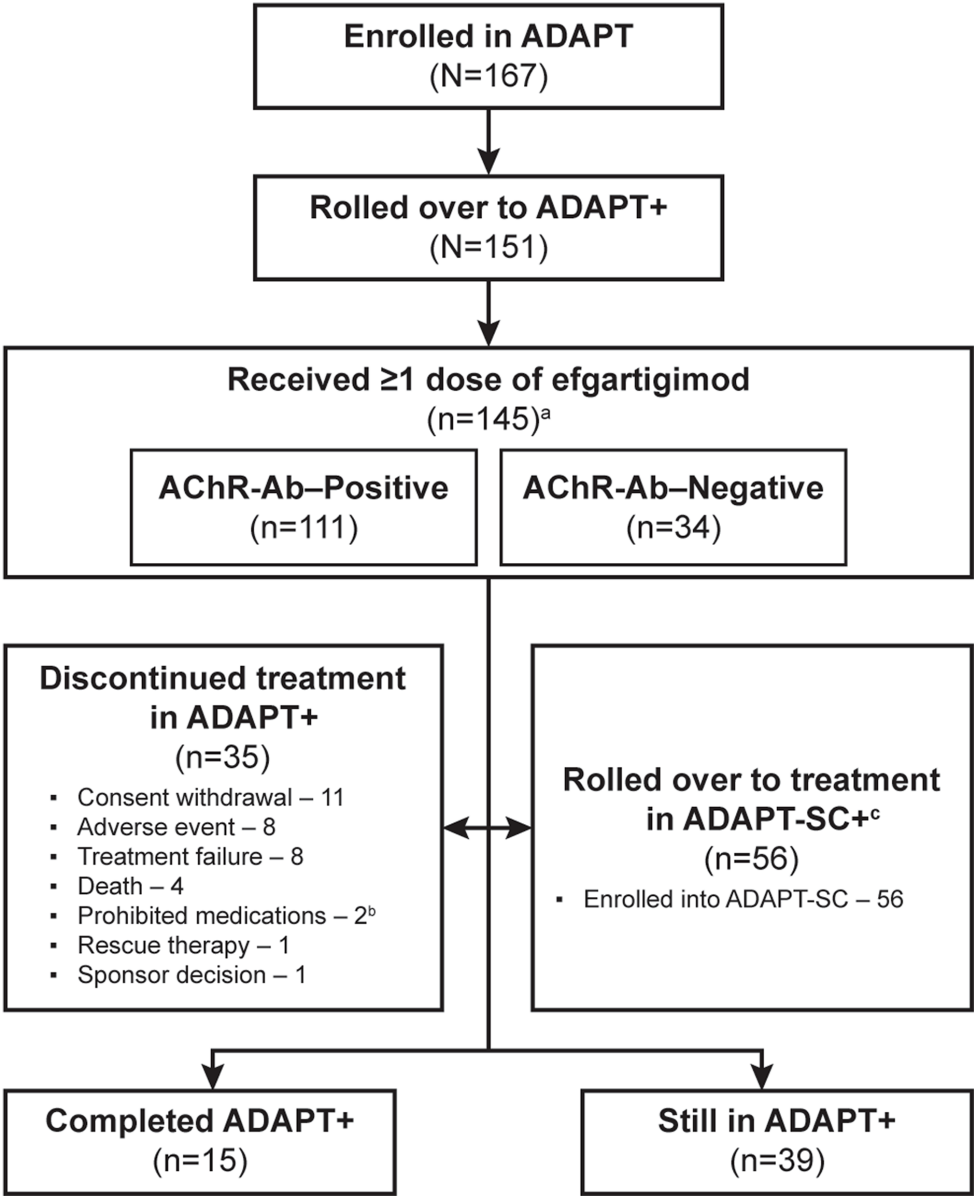
Study participant disposition. AChR-Ab, acetylcholine receptor antibody. ^a^ 6 participants who rolled over to ADAPT+ did not require subsequent treatment cycles based on the defined clinical evaluation criteria. ^b^ 1 of the 2 participants who had been discontinued due to using prohibited medications later died; therefore, this participant was not considered as having discontinued treatment due to death. ^c^ Eligibility for ADAPT-SC+ study was based on completion of at least 1 cycle of treatment and at least 1 year of ADAPT+ study and had started year 2.

As of the interim data cut-off date of January 31, 2022, 151/167 (90%) of participants enrolled in ADAPT had rolled over into ADAPT+, and 145 had received ≥1 dose of efgartigimod (Figure 2). Six participants who rolled over to ADAPT+ did not require subsequent treatment cycles based on the defined clinical evaluation criteria. Of those in ADAPT+, 77 (50.1%) participants had received efgartigimod in ADAPT and 68 (45.0%) had received placebo. A total of 35 (24.1%) participants discontinued over the 3-year study period; the most-common reasons were consent withdrawal (11 [7.6%]), AE (8 [5.5%]), and treatment failure (8 [5.5%]). Other reasons are detailed in Figure 2. Additionally, 56 (38.6%) participants in ADAPT+ elected to roll over into the ADAPT-SC+ study.

Participant characteristics for the study population were reported for the ADAPT study and were representative of a broad gMG patient population with respect to age, sex, disease severity, and concomitant gMG treatments (18). In the overall population, 111 (76.6%) participants were AChR-Ab+ and 34 (23.4%) were AChR-Ab−; 4 (2.8%) were AChR-Ab− and MuSK-Ab+. Demographics and baseline disease characteristics (Table 1) were also similar among the AChR-Ab+, AChR-Ab–, and overall populations.

**Table 1:** Demographics and baseline characteristics (safety analysis set).

	AChR-Ab+ population (*n* = 111)	AChR-Ab− population (*n* = 34)	Overall population (*n* = 145)
Age in years, mean (SD)	47.1 (15.5)	46.7 (12.2)	47.0 (14.8)
≥65 years, *n* (%)	18 (16.2)	3 (8.8)	21 (14.5)
Sex at birth, *n* (%)			
Female	75 (67.6)	28 (82.4)	103 (71.0)
Male	36 (32.4)	6 (17.6)	42 (29.0)
Race, *n* (%)			
American Indian, Alaska native	2 (1.8)	0	2 (1.4)
Asian	8 (7.2)	3 (8.8)	11 (7.6)
Black	3 (2.7)	2 (5.9)	5 (3.4)
White	97 (87.4)	29 (85.3)	126 (86.9)
Multiple	1 (0.9)	0	1 (0.7)
Years since diagnosis, mean (SD)	9.7 (7.9)	9.9 (9.1)	9.7 (8.2)
MGFA Class at screening, *n* (%)			
Class II	44 (39.6)	11 (32.3)	55 (38.0)
Class III	63 (56.7)	23 (67.6)	86 (59.3)
Class IV	4 (3.6)	0	4 (2.8)
Total MG-ADL score, mean (SD)	9.5 (3.1)	10.7 (3.4)	9.8 (3.2)
MG-ADL score category, *n* (%)			
Score <5	1 (0.9)	0	1 (0.7)
Score 5–7	32 (28.8)	7 (20.6)	39 (26.9)
Score 8–9	24 (21.6)	6 (17.6)	30 (20.7)
Score ≥10	54 (48.6)	21 (61.8)	75 (51.7)
Total QMG score, mean (SD)	15.3 (5.7)	15.8 (6.0)	15.4 (5.7)
Concomitant gMG treatment, *n* (%)			
Any NSIST	67 (60.4)	22 (64.7)	89 (61.4)
Any steroid	85 (76.6)	26 (76.5)	111 (76.6)
No steroid	26 (23.4)	8 (23.5)	34 (23.4)
Anti–MuSK-Ab positive, *n* (%)	0	4 (11.8)	4 (2.8)
Thymectomy, *n* (%)	68 (61.3)	17 (50.0)	85 (58.6)

AChR-Ab+, acetylcholine receptor antibody–positive; AChR-Ab−, acetylcholine receptor antibody–negative; gMG, generalized myasthenia gravis; MG-ADL, Myasthenia Gravis-Activities of Daily Living; MGFA, Myasthenia Gravis Foundation of America; MuSK-Ab, muscle-specific kinase antibody; NSIST, nonsteroidal immunosuppressive therapy; QMG, Quantitative Myasthenia Gravis.

### 3.2 Long-term safety and tolerability

Participants received a maximum of 17 treatment cycles. Mean duration of treatment plus follow-up was 548 days, corresponding to 217.6 participant-years of observation.

**Figure 3:**
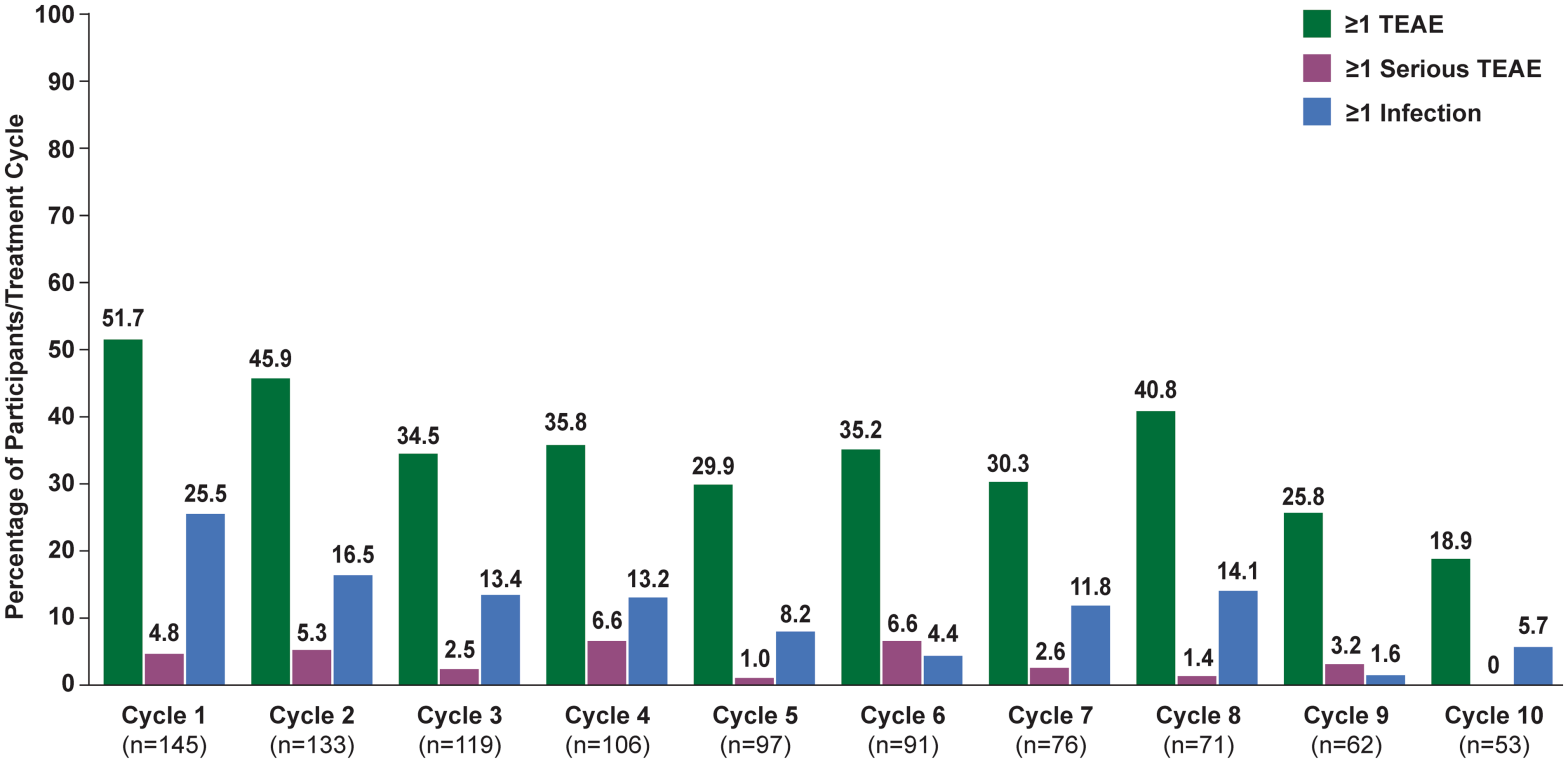
Treatment-emergent adverse events, by cycle (safety analysis set). TEAE, treatment-emergent adverse event. Numbers above bars indicate exact percentage value.

In the overall population, 123 (84.8%) participants reported ≥1 TEAE (Table 2). The most frequently reported TEAEs were headache (36 [24.8%]), COVID-19 (22 [15.2%]), and nasopharyngitis (20 [13.8%]). Other infections included urinary tract infection (13 [9.0%]), herpes zoster (7 [4.8%]), and upper respiratory tract infection (6 [4.1%]). The majority of infections were mild or moderate in severity. TEAEs that led to study discontinuation occurred in 12 (8.3%) participants. Serious TEAEs occurred in 34 (23.4%) participants. The frequency of reported TEAEs, including infections, did not increase with subsequent treatment cycles (Figure 3). All participants with an SAE of MG worsening or MG crisis received rescue therapy (ie, increased dose of corticosteroids, IVIg, or PLEX).

**Table 2:** Treatment-emergent adverse events (safety analysis set).

		Overall population (*n* = 145)	
	*n* (%)	No. of events	ER^a^ (217.55 PY)
All TEAEs	123 (84.8)	783	3.60
Any serious TEAE^b^	34 (23.4)	52	0.24
Any fatal TEAE	5 (3.4)	5	0.02
Any infections (AESIs)	80 (55.2)	164	0.75
Any infusion-related reaction	15 (10.3)	21	0.10
Any TEAE for which efgartigimod was discontinued	12 (8.3)	14	0.06
Most-common TEAEs (reported in ≥5% of participants)			
Headache	36 (24.8)	98	0.45
COVID-19^c^	22 (15.2)	23	0.11
Nasopharyngitis	20 (13.8)	24	0.11
Diarrhea	14 (9.7)	19	0.09
Urinary tract infection	13 (9.0)	18	0.08
Arthralgia	12 (8.3)	15	0.07
Pyrexia	11 (7.6)	11	0.05
Nausea	9 (6.2)	13	0.06
Hypertension	8 (5.5)	11	0.05
Myasthenia gravis worsening	8 (5.5)	10	0.05
Oropharyngeal pain	8 (5.5)	8	0.04

AESI, adverse event of special interest; ER, event rate; IMP, investigational medicinal product; MG, myasthenia gravis; PY, participant-year; SARS-COV-2, severe acute respiratory syndrome coronavirus 2; TEAE, treatment-emergent adverse event. Safety analysis set included all participants who were exposed to the IMP. ^a^ ER was calculated as number of events per PY of follow-up. ^b^ Serious adverse events reported in >1 participant in the total efgartigimod cohort were MG worsening in 7 (4.8%) participants as well as MG crisis, acute respiratory failure, COVID-19, COVID-19 pneumonia, and pneumonia (all in 2 participants [1.4%] each). ^c^ Includes all preferred terms of COVID-19, COVID-19 pneumonia, coronavirus infection, SARS-COV-2 test positive.

There were 5 serious TEAEs with fatal outcome; none were considered by the investigator to be related to efgartigimod treatment. One was acute myocardial infarction in a 55-year-old man with a history of substantial cardiovascular disease, including atherosclerosis and left coronary stent placed during ADAPT screening period. A 66-year-old woman died of lung carcinoma; she had an existing diagnosis of squamous cell carcinoma from 2016. A 79-year-old man with MG exacerbation and bulbar involvement had *Escherichia coli* pneumonia and developed fatal MG crisis. A 72-year-old woman died at home; her death was unwitnessed but coronary artery atherosclerosis (up to 80%) and cardiomegaly were confirmed at autopsy. A 62-year-old man died due to septic shock following COVID-19 infection. All fatal cases were reviewed by an independent data monitoring committee.

Treatment with efgartigimod did not reduce serum albumin levels and did not increase total cholesterol or low-density lipoprotein (LDL) cholesterol concentrations (Supplementary Figure 1). There were no clinically relevant changes from baseline in hematology or chemistry parameters. Vital sign parameters (ie, heart rate, temperature, and systolic/diastolic blood pressure) did not notably differ from baseline.

### 3.3 Pharmacodynamics and immunogenicity

**Figure 4:**
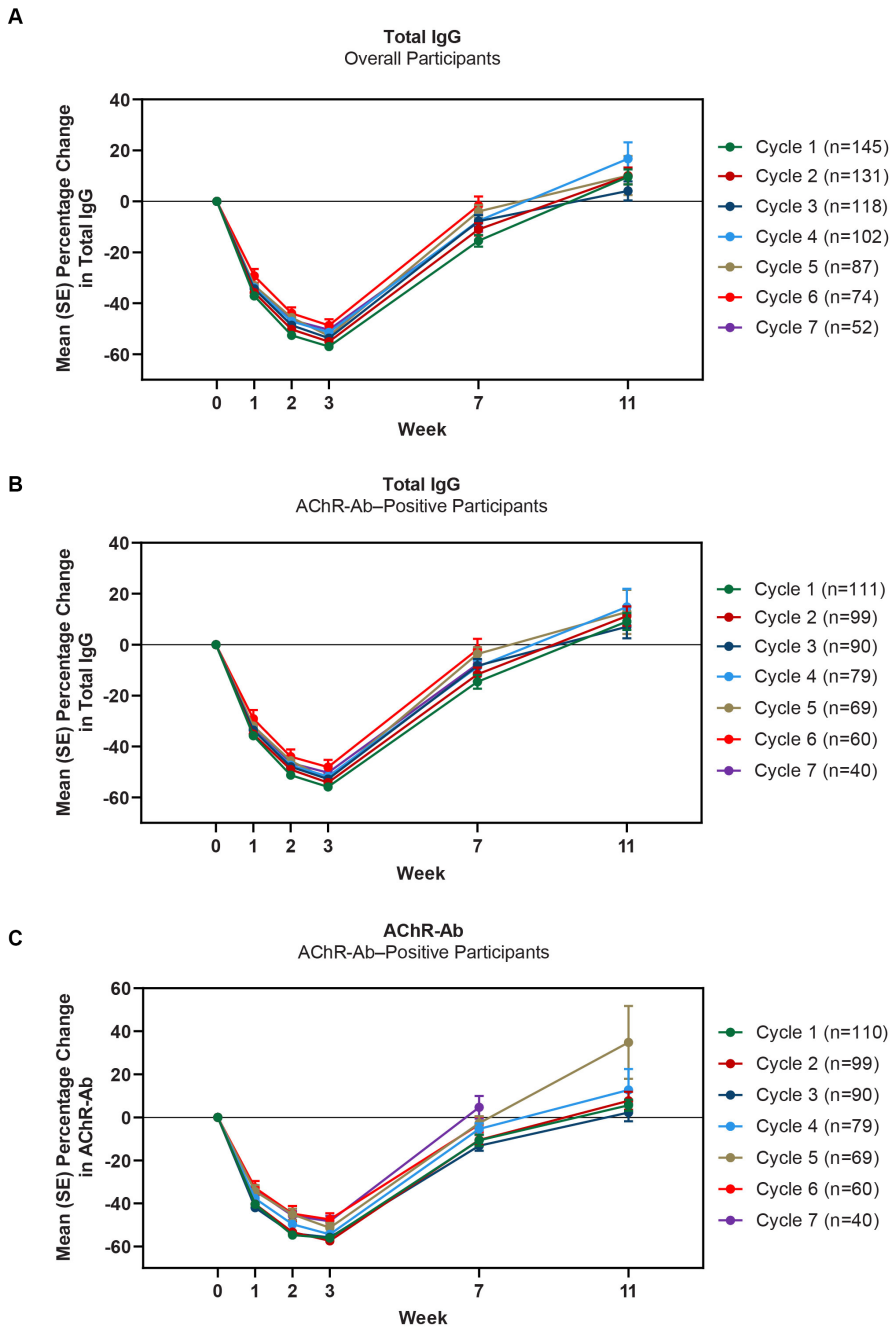
Mean percentage change from cycle baseline in total IgG (overall and AChR-Ab+ populations) and AChR-Ab level. Data for week 11 were not graphed for cycles 6 and 7 because they were unavailable. AChR-Ab, acetylcholine receptor antibody; IgG, immunoglobulin G. **(A)** Mean percentage change from cycle baseline in total IgG (overall). **(B)** Mean percentage change from cycle baseline in total IgG (AChR-Ab+). **(C)** Mean percentage change from cycle baseline in AChR-Ab (AChR-Ab+).

Mean total IgG levels decreased during the 4 infusions and returned to baseline between the week 7 and 11 study visits (Figures 4A,B). The maximum mean percentage reductions from baseline in total IgG levels in AChR-Ab+ participants were observed at week 3 of treatment cycles (ie, immediately before the fourth efgartigimod infusion, though no measurement was taken at week 4, when maximum reductions occurred in ADAPT). In cycle 1, the maximum mean reduction of total IgG level was 55.9% in AChR-Ab+ participants, 60.3% in AChR-Ab− participants, and 57% in the overall population; similar reductions were observed with each subsequent cycle. Additionally, similar reductions were observed in each cycle for AChR-Ab levels in AChR-Ab+ participants (Figure 4C) and for all IgG subtypes (data not shown).

ADA status was evaluable in 138 participants, of whom, 84.1% were ADA-negative. Twenty-two participants (15.9%) were classified as ADA-positive: of these, 18 were positive prior to treatment. The majority of the ADA responses were detected in 1 cycle only, and no apparent increases in ADA titers were observed across treatment cycles. There was also no apparent impact of ADA on pharmacokinetics, pharmacodynamics, safety, or efficacy.

### 3.4 Efficacy

**Figure 5:**
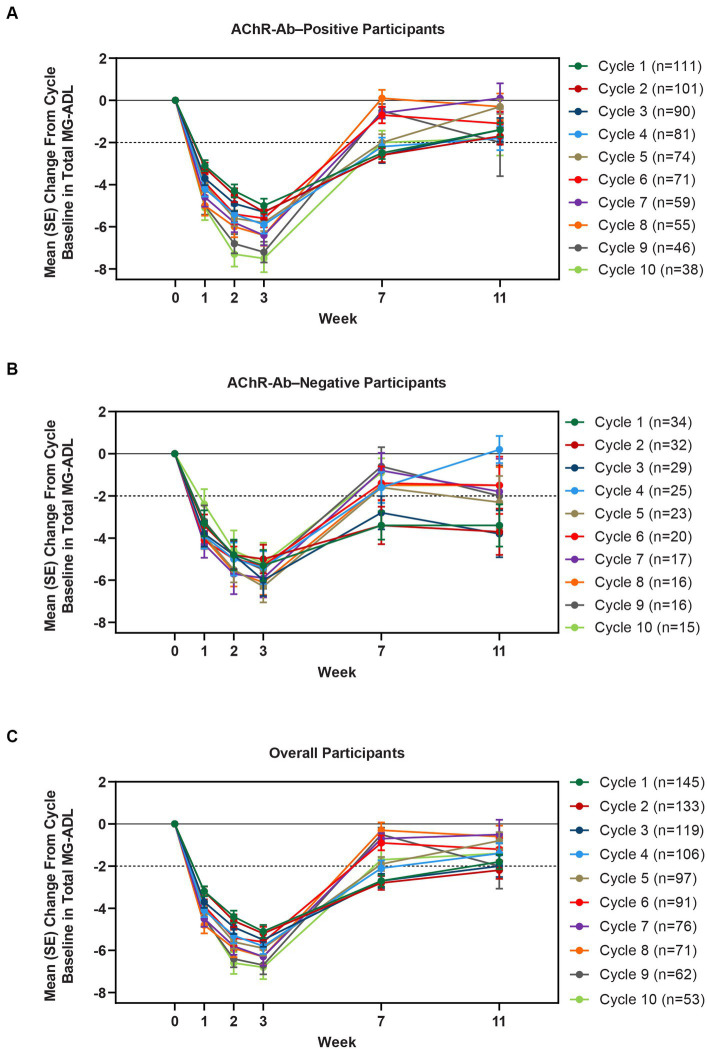
Mean change from cycle baseline in MG-ADL total score, by cycle (AChR-Ab+, AChR-Ab−, and overall populations; safety analysis set). Only time points with ≥3 participants are included. AChR-Ab+, acetylcholine receptor antibody–positive; AChR-Ab−, acetylcholine receptor antibody–negative; CMI, clinically meaningful improvement; MG-ADL, Myasthenia Gravis-Activities of Daily Living. **(A)** Mean (SE) change from cycle baseline in MG-ADL total score (AChR-Ab+). **(B)** Mean (SE) change from cycle baseline in MG-ADL total score (AChR-Ab−). **(C)** Mean (SE) change from cycle baseline in MG-ADL total score (overall population). Dashed line indicates CMI.

**Figure 6:**
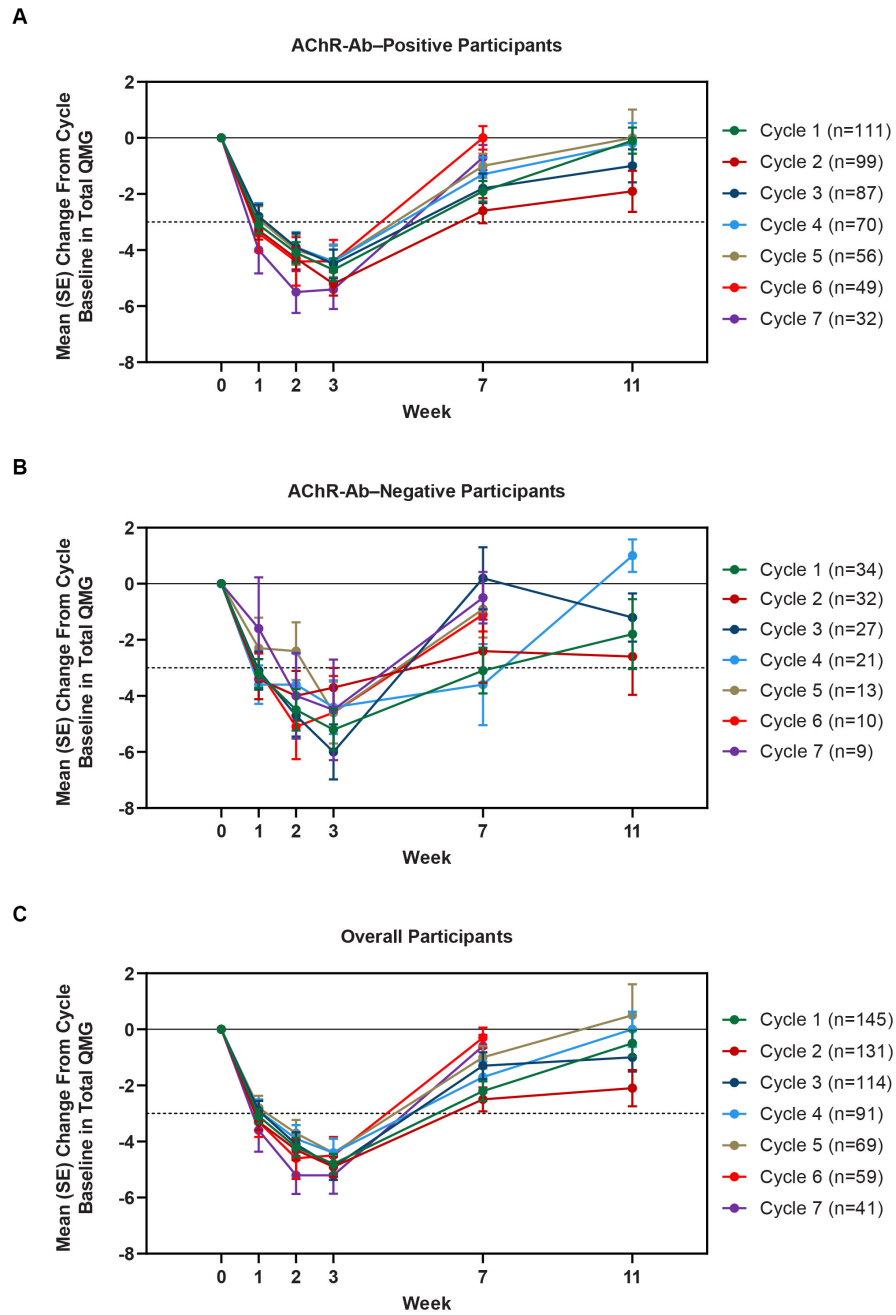
Mean change from cycle baseline in QMG total score, by cycle (AChR-Ab+, AChR-Ab−, and overall populations; safety analysis set). Only time points with ≥3 participants are included. Data for week 11 were not graphed for cycles 6 and 7 because they were unavailable. AChR-Ab+, acetylcholine receptor antibody–positive; AChR-Ab−, acetylcholine receptor antibody–negative; CMI, clinically meaningful improvement; QMG, Quantitative Myasthenia Gravis. **(A)** Mean (SE) change from cycle baseline in QMG total score (AChR-Ab+). **(B)** Mean (SE) change from cycle baseline in QMG total score (AChR-Ab−). **(C)** Mean (SE) change from cycle baseline in QMG total score (overall population). Dashed line indicates CMI.

Clinically meaningful improvements in mean MG-ADL and QMG scores were seen as early as 1 week after the first infusion, and maximal MG-ADL and QMG improvements aligned with total IgG and AChR-Ab reductions. The mean (SE) change from cycle baseline in MG-ADL total score in the AChR-Ab+ population was −5.0 (0.3) at week 3 in cycle 1 and ranged from −5.3 to −7.5 in subsequent cycles (2–10) (Figure 5). For AChR-Ab+ participants, more than 90% had a maximum reduction of ≥2 points (CMI) in MG-ADL total score at any time point in each of the first 10 treatment cycles. Mean (SE) change in QMG score for the AChR-Ab+ population was −4.7 (0.4) at week 3 in cycle 1 and ranged from −4.4 to −5.4 for subsequent cycles (2–7) in the first year (Figure 6). At any time during the 7 cycles for which QMG was measured, 69.4% to 91.3% of AChR-Ab+ participants demonstrated a maximum reduction of ≥3 points (CMI) in QMG total score.

**Figure 7:**
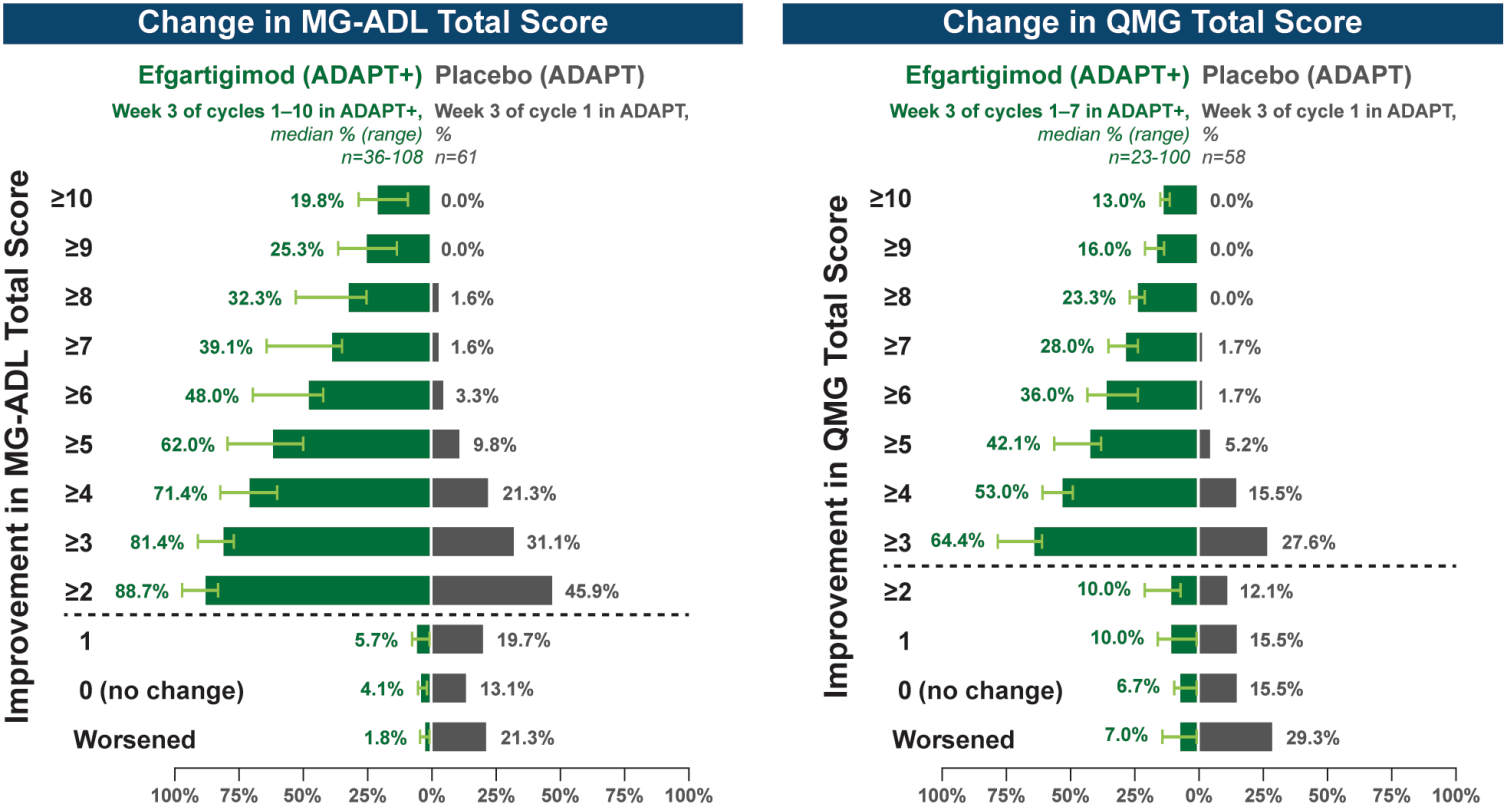
Median cycle proportion of participants with increasing threshold of MG-ADL or QMG improvement, over multiple cycles in ADAPT+ (AChR-Ab+ participants). AChR-Ab+, acetylcholine receptor antibody–positive; CMI, clinically meaningful improvement; MG-ADL, Myasthenia Gravis-Activities of Daily Living; QMG, Quantitative Myasthenia Gravis. Median proportion of AChR-Ab+ participants with increasing thresholds of improvement in MG-ADL (cycle 1 through cycle 10), QMG (cycle 1 through cycle 7), and QMG total score at week 3. Placebo data depicted are from the phase 3 ADAPT study which included 1 cycle (30). Dashed line indicates CMI.

A substantial proportion of participants demonstrated clinical improvements well beyond CMI thresholds (Figure 7), with a median proportion of 25.3% (range: 13.9%–36.1%) achieving at least a 9-point improvement in MG-ADL total score at week 3 and a median proportion of 23.3% (range: 20.6%–26.7%) achieving at least an 8-point improvement in QMG total score at week 3. CMIs were also seen in AChR-Ab− participants, with mean (SE) change from cycle baseline in MG-ADL total score of −5.3 (0.7) at week 3 of cycle 1 (range: −5.0 to −6.3 in subsequent cycles, through cycle 10). Similar results were seen in QMG total score for AChR-Ab− participants, with mean (SE) change from cycle baseline of −5.2 (0.7) at week 3 of cycle 1 (range: −3.7 to −6.0 in subsequent cycles, through cycle 7).

**Figure 8:**
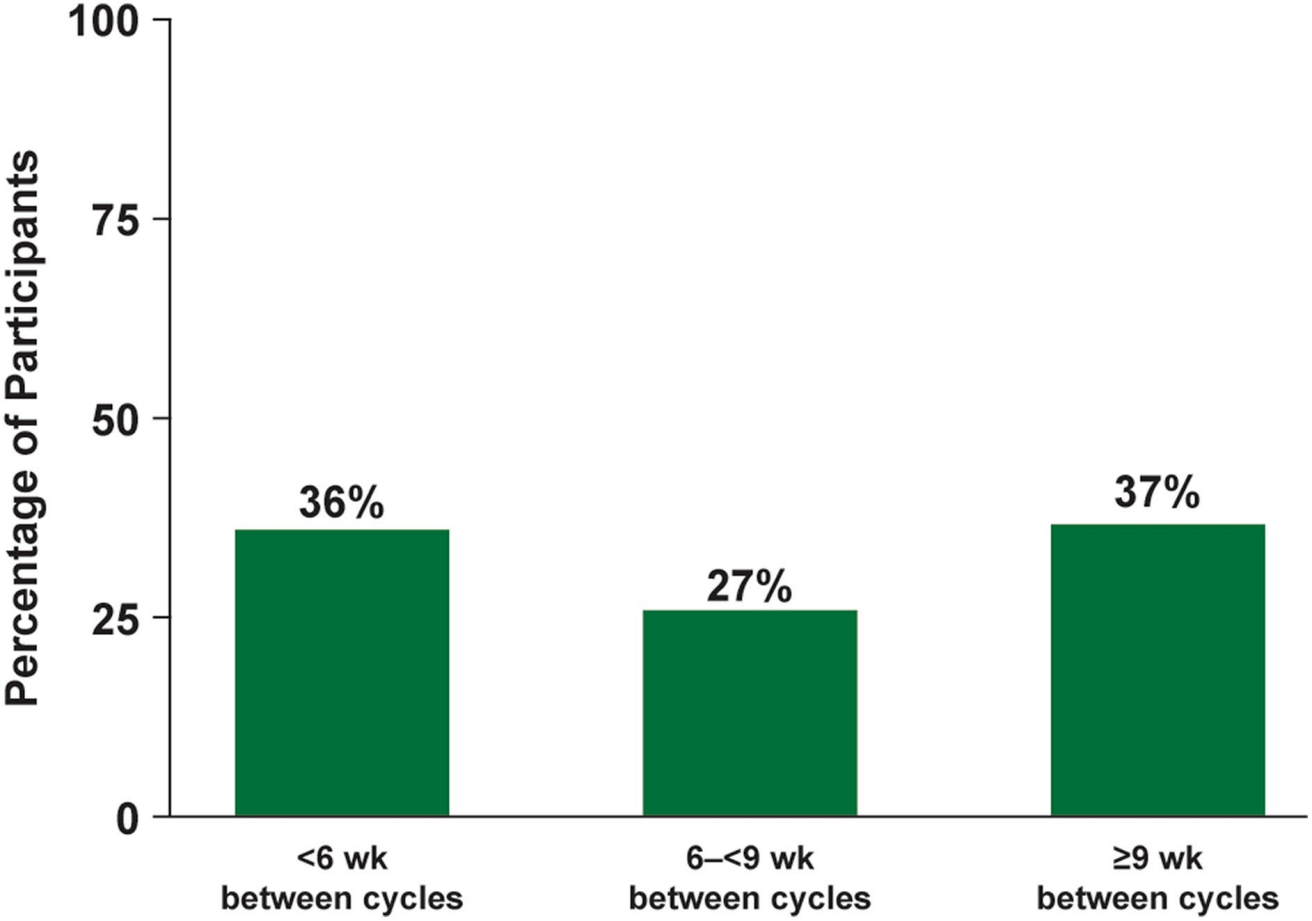
Mean time between cycles in AChR-Ab+ population with ≥1 year of follow-up in ADAPT/ADAPT+ (*n* = 95). AChR-Ab+, acetylcholine receptor antibody–positive. *Post hoc* analysis of mean time between cycles in AChR-Ab+ participants with ≥1 year of follow-up in ADAPT/ADAPT+. For each individual participant, time between the last infusion of preceding cycle and the first infusion of the following cycle was calculated, and average duration was calculated for each participant for all completed cycles.

In a *post hoc* analysis of 95 AChR-Ab+ participants with at least 1 year of combined follow-up in ADAPT and ADAPT+, the annualized mean number of cycles was 4.7 cycles per year (median [range] 5.0 [0.5–7.6]). The average time between treatment cycles (from the last infusion of the previous cycle to the first infusion of the subsequent cycle) was at least 9 weeks in 37% of participants, which equates to approximately 4 cycles per year (Figure 8). This indicates sustained clinical benefit beyond the duration of IgG and AChR-Ab reduction. Additionally, extended duration of clinical improvement that warranted fewer cycles per year was observed, with 24% receiving ≤3 cycles per year, 18% receiving ≤2 cycles per year, and 6% receiving 1 cycle per year.

## 4 Discussion

The ADAPT study demonstrated that FcRn blockade via efgartigimod was well tolerated in patients with gMG and showed the effectiveness of selective IgG reduction, which resulted in clinically meaningful improvements in both function and muscle strength (18). This interim analysis of the open-label extension study provides additional evidence that efgartigimod remained safe and well tolerated, with consistent and repeatable clinical improvements across multiple treatment cycles.

The ADAPT and ADAPT+ studies treated a broad gMG patient population, including those in early and late stages of the disease course, with mild to moderate disease severity, and regardless of autoantibody status (AChR-Ab+ and AChR-Ab− participants). Additionally, the participants were receiving a broad range of commonly used MG treatments, including a cohort receiving only AChE inhibitors. In ADAPT+, efgartigimod was well tolerated and demonstrated a safety profile consistent with the ADAPT study, which was maintained over multiple treatment cycles (up to a maximum of 17 cycles) and 217.6 participant-years of observation. Repeated treatment cycles with efgartigimod did not result in an increase of existing AEs or the appearance of new AEs.

Recent literature suggests incidence of severe infections is increased in patients with gMG, which is potentially due to utilization of broadly immunosuppressive treatments (29). In ADAPT+, the proportion of participants experiencing an infection did not increase with subsequent cycles. Similar to results from the ADAPT study, the majority of infections observed with long-term efgartigimod treatment in ADAPT+ were mild or moderate in severity. Although not definitive, these data are reassuring and reflect efgartigimod’s mechanism of action of selective IgG reduction, which leads to substantial but incomplete IgG reduction without altering other immunoglobulins. FcRn blockade does not inhibit IgG production, therefore allowing the immune system to mount a cellular and humoral response upon antigen challenge (16); in ADAPT+, antigen-specific IgG responses were observed in participants who received T-cell–dependent and T-cell–independent vaccines, indicating they retained the ability to mount an immune response (17).

Reductions in total IgG, IgG subtypes, and AChR antibodies were consistent with ADAPT and were repeatable across multiple cycles in ADAPT+. The observed mean reduction in IgG and IgG subtypes were similar, regardless of antibody status. Additionally, whereas some FcRn inhibitors have been shown to reduce serum albumin levels and subsequently increase cholesterol levels (14), treatment with efgartigimod did not decrease mean serum albumin levels or increase mean LDL or total cholesterol levels. This may be in part due to efgartigimod being an Fc fragment, the natural binding ligand to FcRn, and thus does not interfere with the albumin binding site (13).

CMIs were shown in both MG-ADL and QMG total scores across multiple treatment cycles, with improvements occurring as early as 1 week following the first infusion of a treatment cycle. At any time point in the first 10 treatment cycles, more than 90% of AChR-Ab+ participants achieved CMI in MG-ADL, while in the 7 cycles in which QMG was measured, the percentage of AChR-Ab+ participants who achieved CMI in QMG total score ranged from 69.4% to 91.3%. Clinical improvements well beyond CMI thresholds were observed in AChR-Ab+ participants across multiple treatment cycles. Maximal mean MG-ADL and QMG improvements aligned with timing and magnitude of mean total IgG and AChR-Ab reductions.

Among AChR-Ab− participants, reduction in total IgG and clinical improvements were similar to those observed in AChR-Ab+ participants. Clinical improvements in AChR-Ab− participants were consistent and repeatable across multiple cycles, with mean change at week 3 from cycle baseline MG-ADL and QMG total scores being greater than the defined threshold for CMI. These data are especially encouraging considering the significant disease burden and high unmet need experienced by AChR-Ab− patients (12, 31).

The individualized dosing approach of ADAPT and ADAPT+, which was developed following consultation with patients and clinicians, allowed for the frequency of treatment cycles to be determined by clinical evaluation of each participant. Participants experienced consistent clinical improvement with each cycle, and in AChR-Ab+ participants with at least 1 year of combined follow-up between ADAPT and ADAPT+, the mean number of annualized cycles in ADAPT/ADAPT+ was 4.7 cycles per year (median [range], 5.0 [0.5–7.6]). Some participants demonstrated sustained duration of clinical improvement, which warranted fewer cycles per year, and supports an individualized treatment approach based on clinical evaluation.

Limitations of this study include its open-label design and the limited number of assessment time points (weeks 0, 1, 2, 3 and then every 30 days during year 1, and every 90 days in years 2 and 3). These time points may not have captured the maximum reductions in MG-ADL, QMG, and total IgG/AChR-Ab levels that were observed to occur at week 4 in ADAPT (ie, 1 week after the last infusion of a cycle).

Overall, these interim results of ADAPT+ support the long-term safety, tolerability, and efficacy of efgartigimod, an Fc fragment, for the treatment of patients with gMG. Rapid onset of action, along with significant and repeatable improvements in activities of daily living and muscle strength, across ≤17 treatment cycles, support a long-term individualized treatment strategy for FcRn blockade in a broad population of patients with gMG.

## Data availability statement

The datasets presented in this article are not readily available but can be requested by qualified researchers who engage in rigorous independent scientific research and can be provided after review and approval of a research proposal and statistical analysis plan and execution of a data sharing agreement. Data requests can be submitted at any time and the data will be accessible for 12 months. argenx is committed to responsible data sharing regarding the clinical trials they fund. Included in this commitment is access to anonymized individual and trial-level data (analysis datasets), and other information (eg, protocols and clinical study reports), as long as the trials are not part of an ongoing or planned regulatory submission. This includes requests for clinical trial data for unlicensed products and indications. Requests to access the datasets should be directed to ESR@argenx.com.

## Ethics statement

The studies involving humans were approved by institutional review boards, ethics committees, and regulatory agencies. The studies were conducted in accordance with the local legislation and institutional requirements. The participants provided their written informed consent to participate in this study.

## Author contributions

JH: Conceptualization, Investigation, Methodology, Writing – review & editing. VB: Data curation, Writing – review & editing. TV: Data curation, Writing – review & editing. CK: Data curation, Writing – review & editing. SP: Data curation, Writing – review & editing. JB: Data curation, Writing – review & editing. HM: Data curation, Writing – review & editing. AM: Data curation, Writing – review & editing. SB: Data curation, Writing – review & editing. MP: Data curation, Writing – review & editing. AG: Conceptualization, Methodology, Writing – review & editing. BH: Conceptualization, Methodology, Writing – review & editing. SS: Conceptualization, Data curation, Methodology, Writing – review & editing. CT’j: Conceptualization, Data curation, Methodology, Writing – review & editing. KU: Data curation, Writing – review & editing. JV: Data curation, Writing – review & editing. RM: Data curation, Writing – review & editing.

## Supplementary material

SUPPLEMENTARY FIGURE 1 (A) Mean (SD) total albumin levels; (B) Mean (SD) total cholesterol levels; (C) Mean (SD) LDL cholesterol levels. ^a^Reference values are based on Kratz A, et al. *N Engl J Med*. (2004) 351:1548-63.
