# Supplementary material for: Long-term safety, tolerability, and efficacy of efgartigimod (ADAPT+): interim results from a phase 3 open-label extension study in participants with generalized myasthenia gravis
Source: Front Neurol. 2024 Jan 17;14:1284444. doi: 10.3389/fneur.2023.1284444 (PMC10842202; doi:10.3389/fneur.2023.1284444)
Supplement: Supplementary file 1 [file Table_1.DOCX]

**Supplemental Table 1: Inclusion and Exclusion Criteria**

| **Inclusion Criteria** | The following inclusion criteria must have been met for inclusion in this study:  1. Participants had the ability to understand the requirements of the study, provided written informed consent, and could comply with the study protocol procedures.  2. Participants who participated in the pivotal ADAPT study and were eligible for rollover, ie:   - Participants who reached end of study at day 182 in the pivotal ADAPT study - Participants who needed retreatment in the ADAPT study but could not complete a treatment cycle within the time frame of that study could immediately roll over into this study to receive treatment with efgartigimod - Participants who discontinued early from randomized treatment for reasons other than pregnancy, rescue therapy, or an (S)AE in the pivotal ADAPT study may be offered the option to roll over into this study - Participants who had a temporary interruption from randomized treatment in the pivotal ADAPT study may be offered the option to roll over into this study - Participants were required to be on a stable dose of their concomitant gMG treatment prior to study entry baseline. The concomitant gMG treatment was limited to AChE inhibitors, steroids, and NSISTs (eg, azathioprine, methotrexate, cyclosporine, tacrolimus, mycophenolate mofetil, and cyclophosphamide). |
| --- | --- |
| **Exclusion Criteria** | Participants did not roll over into this study if any of the following criteria were met:  1. Participants who discontinued early from the pivotal ADAPT study or participants who discontinued early from randomized treatment for pregnancy or rescue reasons or an SAE that was likely to result in a life-threatening situation or pose a serious safety risk.  2. Pregnant and lactating women, and those intending to become pregnant during the study or within 90 days after the last dosing. Women of childbearing potential must have had a negative urine pregnancy test at study entry baseline.  3. Male participants who were sexually active and did not intend to use effective methods of contraception (as mentioned above) during the study or within 90 days after the last dosing or male participants who planned to donate sperm during the study or within 90 days after the last dosing.  4. Participants with known hepatitis B virus, hepatitis C virus, or human immunodeficiency virus seropositivity.  5. Participants with known autoimmune disease other than MG (eg, autoimmune thyroiditis, rheumatoid arthritis) that would interfere with an accurate assessment of clinical symptoms.  6. Participants with clinical evidence of other significant disease or participants who underwent a recent major surgery, which could confound the results of the study or put the participant at undue risk. Participants with renal/hepatic function impairment could have been included.  7. Participants with known medical history of hypersensitivity to any of the ingredients of efgartigimod |

AChE, acetylcholinesterase; AE, adverse event; MG, myasthenia gravis; NSIST, nonsteroidal immunosuppressive therapy; SAE, serious adverse event
