# Supplementary material for: Long-term safety, tolerability, and efficacy of efgartigimod (ADAPT+): interim results from a phase 3 open-label extension study in participants with generalized myasthenia gravis
Source: Front Neurol. 2024 Jan 17;14:1284444. doi: 10.3389/fneur.2023.1284444 (PMC10842202; doi:10.3389/fneur.2023.1284444)
Supplement: Supplementary file 2 [file Table_2.DOCX]

**Supplemental Table 2: ADAPT+ Study Group**

| **Investigator** | **Site** | **Country** |
| --- | --- | --- |
| Jan De Bleecker | AZ Sint-Lucas Gent, Department of Neurology | Belgium |
| Annelien De Pue | AZ Sint-Lucas Gent, Department of Neurology | Belgium |
| Rudolf Mercelis | Antwerp University Hospital, Department of Neurology | Belgium |
| Jonathan Baets | Antwerp University Hospital, Department of Neurology | Belgium |
| Vera Bril | Toronto General Hospital, University Health Network | Canada |
| Hans Katzberg | Toronto General Hospital, University Health Network | Canada |
| Jana Junkerova | Neurology Clinic, Faculty Hospital Ostrava | Czech Republic |
| Jana Horakova | Neurology Clinic, Faculty Hospital Ostrava | Czech Republic |
| Katarina Reguliova | Neurology Clinic, Faculty Hospital Ostrava | Czech Republic |
| Michaela Tyblova | Department of Neurology and Center of Clinical Neuroscience First Faculty of Medicine, Charles University and General University Hospital in Prague | Czech Republic |
| Iveta Novakova | Department of Neurology and Center of Clinical Neuroscience First Faculty of Medicine, Charles University and General University Hospital in Prague | Czech Republic |
| Jiri Pitha | Department of Neurology and Center of Clinical Neuroscience First Faculty of Medicine, Charles University and General University Hospital in Prague | Czech Republic |
| Stanislav Vohanka | Fakultní nemocnice Brno | Czech Republic |
| Tomas Horak | Fakultní nemocnice Brno | Czech Republic |
| Magda Horakova | Fakultní nemocnice Brno | Czech Republic |
| Andreas Meisel | Charité - Universitätsmedizin Berlin, Department of Neurology, NeuroCure Clinical Research Center | Germany |
| Sarah Hoffmann | Charité - Universitätsmedizin Berlin, Department of Neurology, NeuroCure Clinical Research Center | Germany |
| Lea Gerischer | Charité - Universitätsmedizin Berlin, Department of Neurology, NeuroCure Clinical Research Center | Germany |
| Frauke Stascheit | Charité - Universitätsmedizin Berlin, Department of Neurology, NeuroCure Clinical Research Center | Germany |
| Anne Lesemann | Charité - Universitätsmedizin Berlin, Department of Neurology, NeuroCure Clinical Research Center | Germany |
| Hannah Pressler | Charité - Universitätsmedizin Berlin, Department of Neurology, NeuroCure Clinical Research Center | Germany |
| Maike Krause | Charité - Universitätsmedizin Berlin, Department of Neurology, NeuroCure Clinical Research Center | Germany |
| John Vissing | Copenhagen Neuromuscular Center, 8077, Rigshospitalet, University of Copenhagen | Denmark |
| Joan Hoejgaard | Copenhagen Neuromuscular Center, 8077, Rigshospitalet, University of Copenhagen | Denmark |
| Mads Stemmerik | Copenhagen Neuromuscular Center, 8077, Rigshospitalet, University of Copenhagen | Denmark |
| Nanna Witting | Copenhagen Neuromuscular Center, 8077, Rigshospitalet, University of Copenhagen | Denmark |
| Nicolai Preisler | Copenhagen Neuromuscular Center, 8077, Rigshospitalet, Universitu of Copenhagen | Denmark |
| Temur Margania | Ltd New Hospitals, Department of Neurology | Georgia |
| Dali Kankava | Ltd New Hospitals, Department of Neurology | Georgia |
| Khatuna Sitchinava | Ltd New Hospitals, Department of Neurology | Georgia |
| Nona Buchashvili | Ltd New Hospitals, Department of Neurology | Georgia |
| Roman Shakarishvili | Ltd Petre Sarajishvili Institute of Neurology | Georgia |
| Nana Kvirkvelia | Ltd Petre Sarajishvili Institute of Neurology | Georgia |
| Elene Nebadze | Ltd Petre Sarajishvili Institute of Neurology | Georgia |
| Alexander Tsiskaridze | Department of Neurology, Tbilisi State University, Pineo Medical Ecosystem, Tbilisi | Georgia |
| Tamar Vashadze | Department of Neurology, Tbilisi State University, Pineo Medical Ecosystem, Tbilisi | Georgia |
| Ani Antia | Department of Neurology, Tbilisi State University, Pineo Medical Ecosystem, Tbilisi | Georgia |
| Lela Tavzarashvili | Department of Neurology, Tbilisi State University, Pineo Medical Ecosystem, Tbilisi | Georgia |
| Csilla Rózsa | Jahn Ferenc Dél-pesti Hospital Budapest Department of Neurology | Hungary |
| David Bors | Jahn Ferenc Dél-pesti Hospital Budapest Department of Neurology | Hungary |
| Renato Mantegazza | lstituto Neurologico Carlo Besta, I.R.C.C.S U.O. Neuroimmunologia e malattie neuromuscolari | Italy |
| Carlo Antozzi | lstituto Neurologico Carlo Besta, I.R.C.C.S U.O. Neuroimmunologia e malattie neuromuscolari | Italy |
| Silvia Bonanno | lstituto Neurologico Carlo Besta, I.R.C.C.S U.O. Neuroimmunologia e malattie neuromuscolari | Italy |
| Rita Frangiamore | lstituto Neurologico Carlo Besta, I.R.C.C.S U.O. Neuroimmunologia e malattie neuromuscolari | Italy |
| Riccardo Giossi | lstituto Neurologico Carlo Besta, I.R.C.C.S U.O. Neuroimmunologia e malattie neuromuscolari | Italy |
| Lorenzo Maggi | lstituto Neurologico Carlo Besta, I.R.C.C.S U.O. Neuroimmunologia e malattie neuromuscolari | Italy |
| Fiammetta Vanoli | lstituto Neurologico Carlo Besta, I.R.C.C.S U.O. Neuroimmunologia e malattie neuromuscolari | Italy |
| Francesco Sacca | Federico II University, NSRO Department | Italy |
| Ciara Pane | Federico II University, NSRO Department | Italy |
| Nunzia Cuomo | Federico II University, NSRO Department | Italy |
| Alessio Sarnataro | Federico II University, NSRO Department | Italy |
| Angela Marsili | Federico II University, NSRO Department | Italy |
| Giorgia Puorro | Federico II University, NSRO Department | Italy |
| Giovanni Antonini | Sant'Andrea Hospital, Department of Neuroscience, Mental Health and Sensory Organs, Sapienza University of Rome | Italy |
| Girolamo Alfieri | Sant'Andrea Hospital, Department of Neuroscience, Mental Health and Sensory Organs, Sapienza University of Rome | Italy |
| Laura Fionda | Sant'Andrea Hospital, Department of Neuroscience, Mental Health and Sensory Organs, Sapienza University of Rome | Italy |
| Matteo Garibaldi | Sant'Andrea Hospital, Department of Neuroscience, Mental Health and Sensory Organs, Sapienza University of Rome | Italy |
| Luca Leonardi | Sant'Andrea Hospital, Department of Neuroscience, Mental Health and Sensory Organs, Sapienza University of Rome | Italy |
| Stefania Morino | Sant'Andrea Hospital, Department of Neuroscience, Mental Health and Sensory Organs, Sapienza University of Rome | Italy |
| Fiammetta Vanoli | Sant'Andrea Hospital, Department of Neuroscience, Mental Health and Sensory Organs, Sapienza University of Rome | Italy |
| Akiyuki Uzawa | Chiba University Hospital, Department of Neurology | Japan |
| Yukiko Ozawa | Chiba University Hospital, Department of Neurology | Japan |
| Yosuke Onishi | Chiba University Hospital, Department of Neurology | Japan |
| Manato Yasuda | Chiba University Hospital, Department of Neurology | Japan |
| Kimiaki Utsugisawa | Hanamaki General Hospital | Japan |
| Yuriko Nagane | Hanamaki General Hospital | Japan |
| Yasushi Suzuki | Sendai Medical Center, Department of Neurology | Japan |
| Kenichi Tsukita | Sendai Medical Center, Department of Neurology | Japan |
| Genya Watanabe | Sendai Medical Center, Department of Neurology | Japan |
| Masanori Takahashi | Osaka University Hospital, Department of Neurology | Japan |
| Tomoya Kubota | Osaka University Hospital, Department of Neurology | Japan |
| Tatsusada Okuno | Osaka University Hospital, Department of Neurology | Japan |
| Makoto Kinoshita | Osaka University Hospital, Department of Neurology | Japan |
| Daisuke Yamamoto | Sapporo Medical University Hospital, Division of Neurology | Japan |
| Shin Hisahara | Sapporo Medical University Hospital, Division of Neurology | Japan |
| Kazuna Ikeda | Sapporo Medical University Hospital, Division of Neurology | Japan |
| Tomihiro Imai | Sapporo Medical University Hospital, Division of Neurology | Japan |
| Masayuki Masuda | Tokyo Medical University Hospital, Department of Neurology | Japan |
| Nobuhiro Ido | Tokyo Medical University Hospital, Department of Neurology | Japan |
| Makiko Kobayashi | Tokyo Medical University Hospital, Department of Neurology | Japan |
| Yoshihiko Okubo | Tokyo Medical University Hospital, Department of Neurology | Japan |
| Takamichi Sugimoto | Hiroshima City Hiroshima Citizens Hospital | Japan |
| Hiroyuki Naito | Hiroshima City Hiroshima Citizens Hospital | Japan |
| Naoko Mine | Hiroshima City Hiroshima Citizens Hospital | Japan |
| Eiichi Nomura | Hiroshima City Hiroshima Citizens Hospital | Japan |
| Takemori Yamawaki | Hiroshima City Hiroshima Citizens Hospital | Japan |
| Johannes Verschuuren | Leids Universitair Medisch Centrum (LUMC) | Netherlands |
| Kevin Keene | Leids Universitair Medisch Centrum (LUMC) | Netherlands |
| Annabel Maria Ruiter | Leids Universitair Medisch Centrum (LUMC) | Netherlands |
| Martijn R. Tannemaat | Leids Universitair Medisch Centrum (LUMC) | Netherlands |
| Linda Remijn | Leids Universitair Medisch Centrum (LUMC) | Netherlands |
| Malgorzata Bilinska | University Clinical Center, Department of Adult Neurology | Poland |
| Marek Halas | University Clinical Center, Department of Adult Neurology | Poland |
| Andrzej Szczudlik | Krakow Academy of Neurology, Center of Clinical Neurology | Poland |
| Grazyna Zwolinska | Krakow Academy of Neurology, Center of Clinical Neurology | Poland |
| Anna Kostera-Pruszczyk | Medical University of Warsaw, Department of Neurology | Poland |
| Aleksandra Golenia | Medical University of Warsaw, Department of Neurology | Poland |
| Ewa Sobieszczuk | Medical University of Warsaw, Department of Neurology | Poland |
| Piotr Szczudlik | Medical University of Warsaw, Department of Neurology | Poland |
| Lech Szczechowski | NZOZ Wielospecjalistyczna Poradnia Lekarska "Synapsis" | Poland |
| Marek Smilowski | NZOZ Wielospecjalistyczna Poradnia Lekarska "Synapsis" | Poland |
| Irina Poverennova | Samara Regional Clinical Hospital, Department of Neurosurgery | Russia |
| Tatiana Romanova | Samara Regional Clinical Hospital, Department of Neurosurgery | Russia |
| Nadezhda Malkova | Regional Centre for Multiple Sclerosis and Other Autoimmune System Diseases of the Nervous System, State Novosibirsk Regional Clinical Hospital | Russia |
| Denis Korobko | Regional Centre for Multiple Sclerosis and Other Autoimmune System Diseases of the Nervous System, State Novosibirsk Regional Clinical Hospital | Russia |
| Ekaterina Bulatova | Regional Centre for Multiple Sclerosis and Other Autoimmune System Diseases of the Nervous System, State Novosibirsk Regional Clinical Hospital | Russia |
| Ilona Vergunova | Regional Centre for Multiple Sclerosis and Other Autoimmune System Diseases of the Nervous System, State Novosibirsk Regional Clinical Hospital | Russia |
| Stojan Peric | University Clinical Center of Serbia, Neurology Clinic, University of Belgrade, Faculty of Medicine | Serbia |
| Ivana Basta | University Clinical Center of Serbia, Neurology Clinic, University of Belgrade, Faculty of Medicine | Serbia |
| Mirjana Arsenijevic | University Clinical Center of Serbia, Neurology Clinic, University of Belgrade, Faculty of Medicine | Serbia |
| Dragana Lavrnic | University Clinical Center of Serbia, Neurology Clinic, University of Belgrade, Faculty of Medicine | Serbia |
| Vidosava Rakocevic Stojanovic | University Clinical Center of Serbia, Neurology Clinic, University of Belgrade, Faculty of Medicine | Serbia |
| Said Beydoun | University of Southern California, Healthcare Consultation Center II | United States |
| Victoria Cannon | University of Southern California, Healthcare Consultation Center II | United States |
| Leila Darki | University of Southern California, Healthcare Consultation Center II | United States |
| Lise Phan | University of Southern California, Healthcare Consultation Center II | United States |
| Norianne Pimentel | University of Southern California, Healthcare Consultation Center II | United States |
| Ken Fujimura | University of Southern California, Healthcare Consultation Center II | United States |
| Michel Darazi | University of Southern California, Healthcare Consultation Center II | United States |
| Hasnain Arshad | University of Southern California, Healthcare Consultation Center II | United States |
| Mimi Lee | University of Southern California, Healthcare Consultation Center II | United States |
| Patricia Lee | University of Southern California, Healthcare Consultation Center II | United States |
| Bo Leung | University of Southern California, Healthcare Consultation Center II | United States |
| James F. Howard Jr. | The University of North Carolina | United States |
| Manisha Chopra | The University of North Carolina | United States |
| Anahit Mehrabyan | The University of North Carolina | United States |
| Rebecca Traub | The University of North Carolina | United States |
| Tahseen Mozaffar | UCI ALS & Neuromuscular Center, Neuromuscular Clinical Trials Unit | United States |
| Jonathan Cauchi | UCI ALS & Neuromuscular Center, Neuromuscular Clinical Trials Unit | United States |
| Ali A. Habib | UCI ALS & Neuromuscular Center, Neuromuscular Clinical Trials Unit | United States |
| Manisha Kak | UCI ALS & Neuromuscular Center, Neuromuscular Clinical Trials Unit | United States |
| Tuan Vu | University of South Florida | United States |
| Jerrica Farias | University of South Florida | United States |
| Niraja Suresh | University of South Florida | United States |
| Sarah Jones | University of Virginia | United States |
| Ted Burns | University of Virginia | United States |
| Allison Crowell | University of Virginia | United States |
| Matthew Elliott | University of Virginia | United States |
| Goran Rakocevic | University of Virginia | United States |
| Guillermo Solorzano | University of Virginia | United States |
| Tulio Bertorini | Wesley Neurology Clinic | United States |
| Hafiz Elahi | Wesley Neurology Clinic | United States |
| Ratna Bhavaraju-Sanka | University of Texas Health Science Center at San Antonio | United States |
| Carlayne Jackson | University of Texas Health Science Center at San Antonio | United States |
| Shahid Noor-E-Ain | University of Texas Health Science Center at San Antonio | United States |
| Pablo Coss | University of Texas Health Science Center at San Antonio | United States |
| Chafic Karam | Oregon Health & Science University | United States |
| Nizar Chahin | Oregon Health & Science University | United States |
| Thomas Ragole | Oregon Health & Science University | United States |
| Anson Wilks | Oregon Health & Science University | United States |
| Robert Lisak | Wayne State University, Department of Neurology | United States |
| Jacob Rube | Wayne State University, Department of Neurology | United States |
| Mamatha Pasnoor | University of Kansas Medical Center, Department of Neuromuscular Research | United States |
| Richard Barohn | University of Kansas Medical Center, Department of Neuromuscular Research | United States |
| Mazen Dimachkie | University of Kansas Medical Center, Department of Neuromuscular Research | United States |
| Constantine Farmakidis | University of Kansas Medical Center, Department of Neuromuscular Research | United States |
| Duaa Jabari | University of Kansas Medical Center, Department of Neuromuscular Research | United States |
| Omar Jawdat | University of Kansas Medical Center, Department of Neuromuscular Research | United States |
| Jeffrey Statland | University of Kansas Medical Center, Department of Neuromuscular Research | United States |
| Yuebing Li | Cleveland Clinic Foundation, Neuromuscular Center | United States |
| John A Morren | Cleveland Clinic Foundation, Neuromuscular Center | United States |
| Srikanth Muppidi | Department of Neurology and Neurosciences, Stanford School of Medicine | United States |
| Neelam Goyal | Department of Neurology and Neurosciences, Stanford School of Medicine | United States |
| Yuen So | Department of Neurology and Neurosciences, Stanford School of Medicine | United States |
| Michael Pulley | University of Florida Health Science Center Jacksonville | United States |
| Shannon LaBoy | University of Florida Health Science Center Jacksonville | United States |
| Jeffrey Shije | University of Florida Health Science Center Jacksonville | United States |
| Gregory Sahagian | The Neurology Center of Southern California | United States |
| Andrew Bierman | The Neurology Center of Southern California | United States |
| Benjamin Frishberg | The Neurology Center of Southern California | United States |
| J. Peter Heinen | The Neurology Center of Southern California | United States |
| Kinjal Madhav | The Neurology Center of Southern California | United States |
| Tara Quesnell | The Neurology Center of Southern California | United States |
