# Supplementary figures and images for: Long-term safety, tolerability, and efficacy of efgartigimod (ADAPT+): interim results from a phase 3 open-label extension study in participants with generalized myasthenia gravis
Source: Front Neurol. 2024 Jan 17;14:1284444. doi: 10.3389/fneur.2023.1284444 (PMC10842202; doi:10.3389/fneur.2023.1284444)

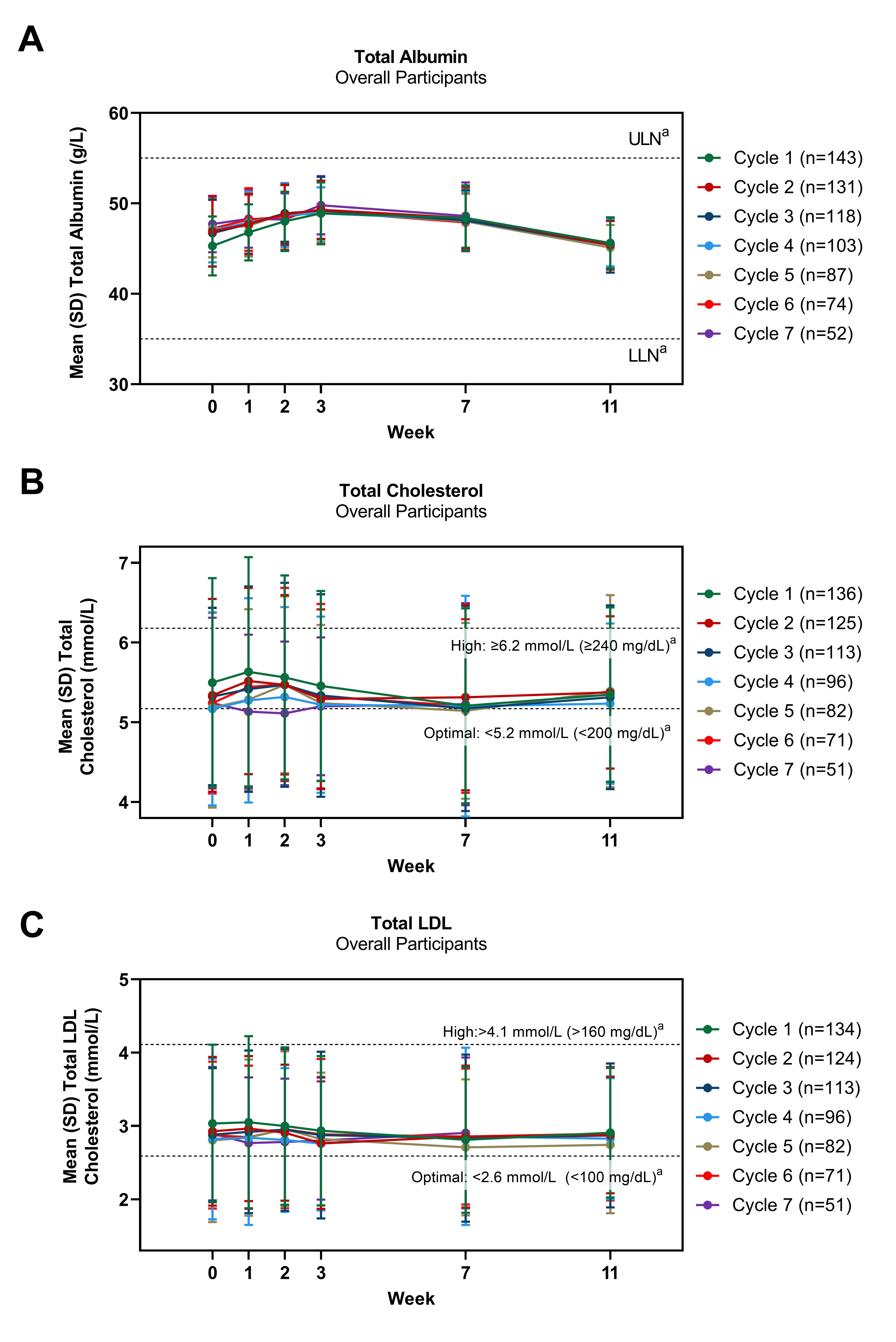

Supplement: SUPPLEMENTARY FIGURE 1 — (A) Mean (SD) total albumin levels; (B) Mean (SD) total cholesterol levels; (C) Mean (SD) LDL cholesterol levels. aReference values are based on Kratz A, et al. N Engl J Med. (2004) 351:1548-63. [file Image_1.jpg]
